# Increased risk of chronic fatigue syndrome following infection: a 17-year population-based cohort study

**DOI:** 10.1186/s12967-023-04636-z

**Published:** 2023-11-11

**Authors:** Hsun Chang, Chien-Feng Kuo, Teng-Shun Yu, Liang-Yin Ke, Chung-Lieh Hung, Shin-Yi Tsai

**Affiliations:** 1 Division of Infectious Diseases, Department of Internal Medicine, MacKay Memorial Hospital, Taipei, Taiwan; 2 Department of Medicine, MacKay Medical College, New Taipei City, 252 Taiwan; 3 Department of Health Policy and Management, Johns Hopkins Bloomberg School of Public Health, Johns Hopkins University, 615 N. Wolfe Street, Baltimore, MD 21205 USA; 4 Management Office for Health Data, China Medical University Hospital, Taichung, Taiwan; 5 Department of Internal Medicine, Taichung Veterans General Hospital, Taichung, Taiwan; 6 Medical Laboratory Science & Biotechnology, Kaohsiung Medical University, Kaohsiung, Taiwan; 7 Division of Cardiology, Departments of Internal Medicine, MacKay Memorial Hospital, Taipei, Taiwan; 8 Institute of Biomedical Sciences, MacKay Medical College, New Taipei City, Taiwan; 9 Department of Laboratory Medicine, MacKay Memorial Hospital, Taipei, 104 Taiwan; 10 Institute of Long-Term Care, MacKay Medical College, New Taipei City, Taiwan

**Keywords:** Chronic fatigue syndrome, Myalgic encephalomyelitis, Pathogen, Infection, Fatigue, Hypothalamic–pituitary–adrenal axis, Autoimmune

## Abstract

**Background:**

Previous serological studies have indicated an association between viruses and atypical pathogens and Chronic Fatigue Syndrome (CFS). This study aims to investigate the correlation between infections from common pathogens, including typical bacteria, and the subsequent risk of developing CFS. The analysis is based on data from Taiwan’s National Health Insurance Research Database.

**Methods:**

From 2000 to 2017, we included a total of 395,811 cases aged 20 years or older newly diagnosed with infection. The cases were matched 1:1 with controls using a propensity score and were followed up until diagnoses of CFS were made.

**Results:**

The Cox proportional hazards regression analysis was used to estimate the relationship between infection and the subsequent risk of CFS. The incidence density rates among non-infection and infection population were 3.67 and 5.40 per 1000 person‐years, respectively (adjusted hazard ratio [HR] = 1.5, with a 95% confidence interval [CI] 1.47–1.54). Patients infected with Varicella-zoster virus, *Mycobacterium tuberculosis*, *Escherichia coli*, *Candida*, *Salmonella*, *Staphylococcus aureus* and influenza virus had a significantly higher risk of CFS than those without these pathogens (*p* < 0.05). Patients taking doxycycline, azithromycin, moxifloxacin, levofloxacin, or ciprofloxacin had a significantly lower risk of CFS than patients in the corresponding control group (*p* < 0.05).

**Conclusion:**

Our population-based retrospective cohort study found that infection with common pathogens, including bacteria, viruses, is associated with an increased risk of developing CFS.

## Introduction

Chronic fatigue syndrome (CFS), also known as myalgic encephalomyelitis (ME), is a mysterious disorder that affects 0.2%–3.48% of the global population, depending on the diagnostic criteria [1, 2]. The condition is characterized by disabling symptoms such as profound fatigue, post-exertional malaise, unrefreshing sleep, cognitive impairment, and orthostatic intolerance that last for at least 6 months, CFS leads to high medical costs but also decreased productivity, resulting in a high economic burden of $17–$24 billion US dollars annually [3–5]. The exact etiology of CFS remains unclear. However, diverse theories have been proposed, including sequelae of infectious diseases, dysregulation of the immune–inflammatory system, and hypothalamic–pituitary–adrenal (HPA) axis dysfunction [3, 6].

Although the exact pathogenesis of CFS warrants additional research, the immune system is believed to play an important role. CFS is associated with elevated levels of proinflammatory cytokines (TNF-α, IFN-γ, IL-6, and IL-1) [7] and dysregulation of immune cells (decreased T-regulatory cells, persistence of autoantibody-generating autoreactive B cells, and reduced cytotoxicity of natural killer cells) [8, 9]. Triggering events—such as pathogen exposure, metal exposure, and environmental factors—initiate immune responses and generate oxidative stress that harms mitochondria [7–10]. The accumulation of stress through these triggering events elicits autoimmunity in genetically predisposed populations and eventually leads to clinical manifestation of diseases [10, 11]. Additionally, inflammation dysregulates the HPA axis [12], resulting in a vicious cycle in which hypocortisolism worsens control over the production of proinflammatory cytokines [8, 9]. Furthermore, circulating cytokines increase blood–brain barrier permeability, activate glial cells, and sensitize neurons to non-noxious stimuli [13]. These manifestations of neuroinflammation may explain the neurological symptoms of CFS, such as fatigue and pain.

Various pathogens have; including viruses and bacteria such as *Borrelia burgdorferi*, *Coxiella burnetii*, *Chlamydia pneumonia*, *Mycoplasma*, *Mycobacterium tuberculosis*, *H. pylori*, *Salmonella*, *Campylobacter*, and *Escherichia coli* have demonstrated the capacity to “trigger” CFS [14–21]. In fact, some patients have reported having a virus-like illness before the onset of CFS [22]. The association between CFS and viruses including Epstein–Barr virus (EBV), cytomegalovirus (CMV), human herpesviruses 6–8, human parvovirus B19, enteroviruses, lentivirus, Ross River virus, and varicella–zoster virus (VZV) has been demonstrated to various degrees [23, 24].

Postinfectious fatigue has been observed in several diseases, such as COVID-19, dengue fever, and influenza [25–30]. Although the link between these pathogens and CFS has not been firmly established, given the hypothesis of the potential triggering role of pathogens in CFS, the association between CFS and many other common pathogens, such as bacteria and fungi, deserves more attention. Accordingly, our study is the first study comprehensively to investigate the relationship between infection with potential pathogens and CFS.

Immunomodulatory properties of some antibiotics—such as macrolides, tetracyclines, and quinolones—have been described and applied in trials to treat various diseases, including multiple sclerosis (MS), chronic obstructive pulmonary disease (COPD), abdominal aneurysm, and cancer [31–34]. Our study also investigated the associations between immunomodulatory antibiotics and the risk of CFS for the further clinical implications.

## Methods

### Data source

The National Health Insurance (NHI) Program was launched in 1995 and currently covers more than 99% of the population in Taiwan. The National Health Insurance Research Database (NHIRD) contains all original data from the NHI program and is updated annually by the National Health Research Institutes. The Longitudinal Generation Tracking Database 2005 (LGTD 2005). It is one of the most comprehensive nationwide population-based databases worldwide, containing a random sample of data for two million individuals recorded in the NHIRD [35–37]. The *International Classification of Diseases, 9th Revision* and *10th Revision, Clinical Modification* (*ICD-9-CM* & *ICD-10-CM*) were used to ascertain diagnoses of diseases. Data analysis was performed at the Health and Welfare Data Center (HWDC), which was established by Taiwan’s Ministry of Health and Welfare (MOHW). This study was approved by the Research Ethics Committee of the China Medical University Hospital [CMUH109-REC2-031(CR-3)] and the Institutional Review Board of MacKay Memorial Hospital (16MMHIS074).

### Study group

In our study, we obtained a cohort with infection and a control group from the longitudinal data set. Patients who received a diagnosis of potential pathogens (*E. coli*, *Staphylococcus aureus*, *Borrelia burgdorferi*, *Mycobacterium tuberculosis*, *Salmonella*, *Chlamydia pneumoniae*, *Orientia tsutsugamushi*, *Mycoplasma*, *Candida*, Enterovirus, VZV, EBV, Influenza virus, Dengue virus) were defined as the cohort with infection. The index date was defined as the first date of diagnosis of a potential pathogen between January 1, 2000, and December 31, 2017. Patients who did not receive a diagnosis of infection with a potential pathogen were defined as the cohort without infection (control group), and their index date was defined as a random date between 2000 and 2017. We excluded patients who had more than one potential pathogen, were aged < 20 years, or had history of pathogens preceding the index date. The potential pathogens examined included VZV (ICD-9-CM: 053; ICD-10-CM: B02), Epstein-Barr virus (ICD-9-CM: 075; ICD-10-CM: B27), *Mycobacterium tuberculosis* (ICD-9-CM: 010–018; ICD-10-CM: A15-A19), *E. coli* (ICD-9-CM: 041.4, 482.82, 008.0, 038.42; ICD-10-CM: J15.5, A04.0-A04.4, B96.2, A41.5), *Candida* (ICD-9-CM: 112; ICD-10-CM: B37), Enterovirus (ICD-9-CM: 008.67, 079.2, 047, 048; ICD-10-CM: B97.1, B34.1, A87.0, B08.4, B08.5), *Salmonella* (ICD-9-CM: 002, 003; ICD-10-CM: A01, A02), *Staphylococcus aureus* (ICD-9-CM: 482.41, 041.11, 038.11; ICD-10-CM: A49.02, A49.01, B95.62, B95.61, B95.8, A41.02, A41.01, J15.212, J15.211, J15.20, J15.21), *Chlamydia pneumoniae* (ICD-9-CM: 483.1; ICD-10-CM: J16.0, P23.1), Influenza virus (ICD-9-CM: 487, 488; ICD-10-CM: J09, J10, J11), *Orientia tsutsugamushi* (ICD-9-CM: 081.2; ICD-10-CM: A75.3), *Mycoplasma* (ICD-9-CM: 483.0, 041.81; ICD-10-CM: A49.3, B96.0, J15.7, J20.0), Dengue virus (ICD-9-CM: 061; ICD-10-CM: A90), and *Borrelia burgdorferi* (ICD-9-CM: 088.81; ICD-10-CM: A69.2).

### Main outcome and confounding variables

The study defined CFS as ICD-9-CM 780.7 and ICD-10-CM G93.3, R53.8. The endpoint of the study was the clinical diagnosis of CFS during the observation period. Patients with CFS before the index date, cancer (ICD-9-CM: 140-208; ICD-10-CM: C00-C97), rheumatoid arthritis(RA) (ICD-9-CM: 714; ICD-10-CM: M06.9), sleep apnea (ICD-9-CM: 327.2, 780.51, 780.53, 780.57; ICD-10-CM: G47.3), narcolepsy (ICD-9-CM: 327.0, 327.1; ICD-10-CM: G47.4), bipolar affective disorders (ICD-9-CM: 296.4-296.8; ICD-10-CM: F31), schizophrenia (ICD-9-CM: 295; ICD-10-CM: F20), delusional disorders (ICD-9-CM: F297; ICD-10-CM: F22), anorexia and bulimia nervosa (ICD-9-CM: 307.1, 307.51; ICD-10-CM: F500, F501, F502), alcohol or other substance abuse (ICD-9-CM: 305; ICD-10-CM: F10, F11, F12, F13, F14, F15, F16, F17, F18, F19), Inflammatory bowel disease (IBD) (ICD-9-CM: 555.0-555.2, 555.9, 556; ICD-10-CM: K50-K51), burn (ICD-9-CM: 940-949; ICD-10-CM: T20-T32), HIV (ICD-9-CM: 042; ICD-10-CM: B20), Systemic Lupus Erythematosus (SLE) (ICD-9-CM: 710.0; ICD-10-CM: M32) and multiple sclerosis (ICD-9-CM: 340; ICD-10-CM: G35) were excluded from the study. The study adjusted for pre-existing comorbidities including hypothyroidism (ICD-9-CM: 243, 244; ICD-10-CM: E02, E03, E89.0), diabetes mellitus (DM) (ICD-9-CM: 243, 244, E03; ICD-10-CM: E02, E03, E89.0), insomnia (ICD-9-CM: 307.42, 327.0, 780.52; ICD-10-CM: G470), depression (ICD-9-CM: 296.2, 296.3, 300.4, 311; ICD-10-CM: F320, F321, F322, F323, F324, F325, F341), anxiety (ICD-9-CM: 300; ICD-10-CM: F41), dementia (ICD-9-CM: 294.1, 294.2; ICD-10-CM: F01-F03), peptic ulcer (ICD-9-CM: 531, 532, 533; ICD-10-CM: K25, K26, K27), obesity (ICD-9-CM: 278; ICD-10-CM: E66), psoriasis (ICD-9-CM: 696; ICD-10-CM: L40), gout (ICD-9-CM: 274; ICD-10-CM: M10), dyslipidemia (ICD-9-CM: 2720, 2721, 2723, 2724; ICD-10-CM: E78.0, E78.1, E78.2, E78.3, E78.4, E78.5), Irritable bowel syndrome (IBS) (ICD-9-CM: 564.1; ICD-10-CM: K58), hepatitis B (ICD-9-CM: 070.2 ~ 070.3; ICD-10-CM: B16, B18.0, B18.1, B19.1), hepatitis C (ICD-9-CM: 070.41, 070.44, 070.51, 070.54,070.7; ICD-10-CM: B17.1, B18.2, B19.2), fibromyalgia (ICD-9-CM: 729.1; ICD-10-CM: M79.7) were also adjusted for in this study, as were antibiotic medications such as doxycycline. (ATC code: J01AA02), Azithromycin (ATC code: J01FA10), Clarithromycin (ATC code: J01FA09), Moxifloxacin (ATC code: J01MA14), Levofloxacin (ATC code: J01MA12) and Ciprofloxacin (ATC code: J01MA02).

### Statistical analysis

The study conducted a data analysis through a retrospective cohort study. The patients were divided into three age groups: less than 40, 40–64, and 65 years or older. The demographic data of the study participants are presented as numbers and percentages for categorical variables and as means and standard deviations (SD) for continuous variables. Differences between variables were determined using the independent Student’s *t* test or Pearson’s chi-square test, as appropriate. Univariate and multivariate Cox proportional hazard models were employed to calculate the hazard ratio (HR), adjusted hazard ratio (aHR), and corresponding 95% confidence interval (CI). The multivariate analysis adjusted for the confounders of age, sex, comorbidities, and medications. Statistical significance was defined as a two-sided *p* value of less than 0.05 in all analyses. All analyses were performed using SAS statistical software (version 9.4; SAS Institute, Cary, NC, USA), and all graphs were plotted using RStudio (version 3.5.2; RStudio Team, Boston, MA, USA).

## Results

**Fig. 1:**
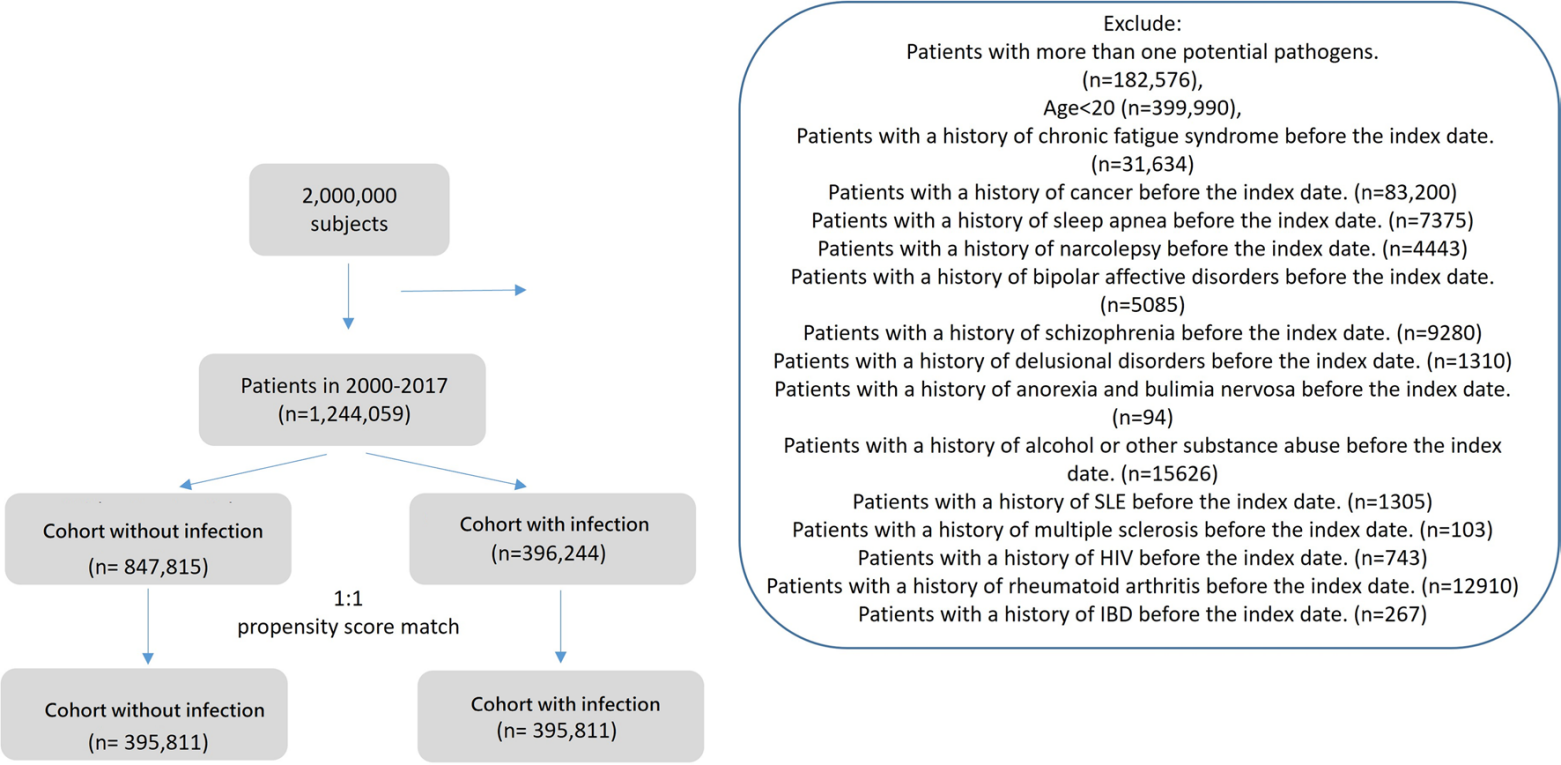
the selection process of the participants

**Fig. 2:**
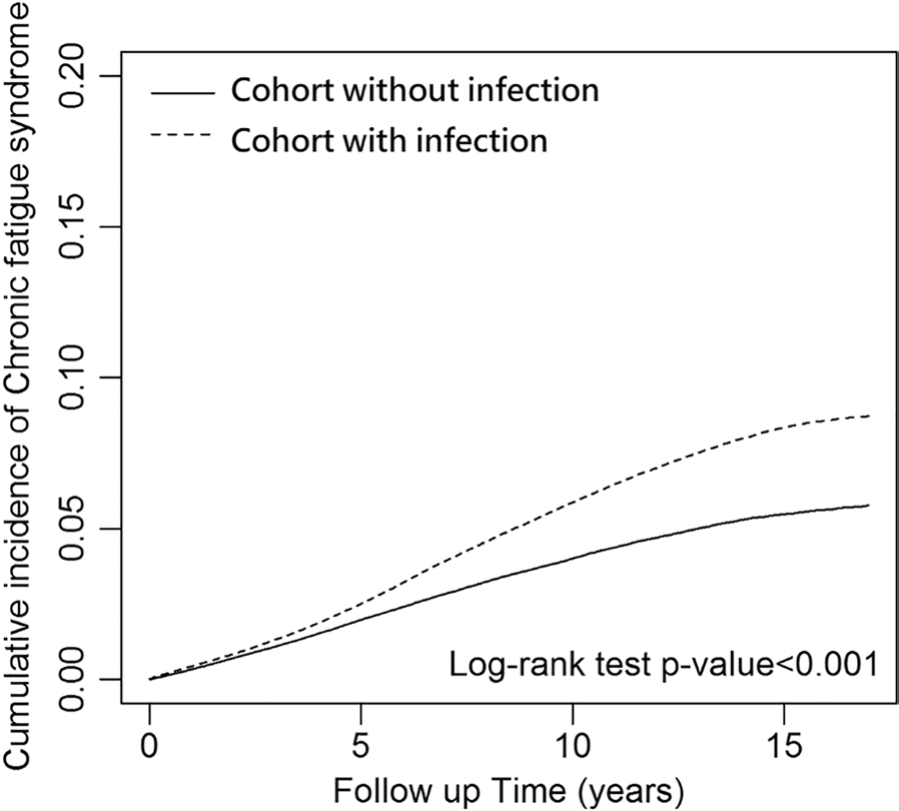
Cumulative incidence of CFS compared between patients with and without pathogens using the Kaplan–Meier method

**Fig. 3:**
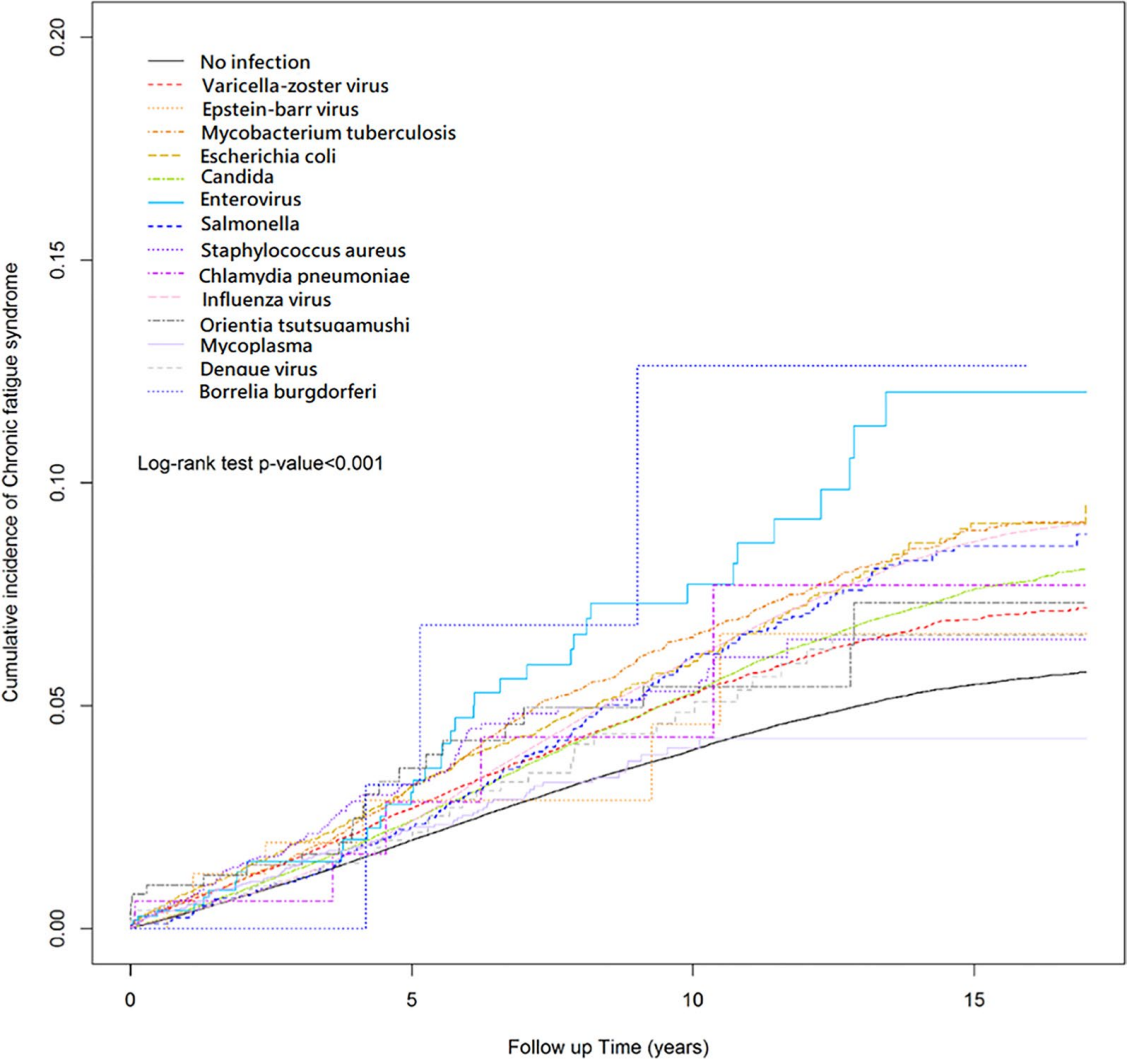
Cumulative incidence of CFS compared between patients infected with different pathogens and without pathogens using the Kaplan–Meier method

Table 1 displays the characteristics of the patients in both infection and control groups. After propensity score matching, there were 395,811 patients in the infection cohort and an equal number of participants in the control group. Figure 1 depicts the patient selection process. The cohort with infection and control group had equivalent proportions of women (58%) and men (42%), and the mean (standard deviation) age of patients in the infection cohort was 45.6 (17.48) years. The three most common baseline comorbidities in the infection cohort were peptic ulcer (20.2%), fibromyalgia (19.95%) and anxiety (14.6%). As indicated in Table 2, the cohort with infection had significantly higher risk of CFS than did the control group (aHR = 1.5, 95% CI 1.47–1.54). Those with VZV (aHR = 1.09, 95% CI 1.04–1.14), *Mycobacterium tuberculosis* (aHR = 1.45, 95% CI 1.34–1.57), *E.coli* (aHR = 1.17, 95% CI 1.05–1.3), *Candida* (aHR = 1.43, 95% CI 1.37–1.48), enterovirus (aHR = 1.86, 95% CI 1.29–2.67), *Salmonella* (aHR = 1.41, 95% CI 1.19–1.67), *Staphylococcus aureus* (aHR = 1.38, 95% CI 1.09–1.74) and influenza virus (aHR = 1.67, 95% CI 1.63–1.71) had significantly higher risk of CFS than did those without these pathogens (*p* < 0.05). The Kaplan–Meier survival curve presented in Fig. 2 illustrates the cumulative incidence of CFS in the two cohorts. Moreover, Fig. 3 provides a graphic representation of the different pathogens. Compared with female patients, male patients had a lower CFS risk. Additionally, patients aged 40–64 years and ≥ 65 years were 1.2 (95% CI 1.17–1.23) and 1.59 (95% CI 1.54–1.65) times more likely, respectively, to develop CFS compared with patients aged < 40 years. Several findings regarding comorbidities and medications emerged from Tables 3 and 4

1. Patients with diabetes mellitus (aHR = 1.05, 95% CI 1.01–1.09), insomnia (aHR = 1.32, 95% CI 1.27–1.37), depression (aHR = 1.07, 95% CI 1.01–1.13), anxiety (aHR = 1.29, 95% CI 1.24–1.33), peptic ulcer (aHR = 1.22, 95% CI 1.18–1.25), gout (aHR = 1.16, 95% CI 1.11–1.21), dyslipidemia (aHR = 1.1, 95% CI 1.06–1.14), irritable bowel hepatitis C (aHR = 1.54, 95% CI 1.39–1.71), or fibromyalgia (aHR = 1.29, 95% CI 1.26–1.33) had a significantly higher risk of CFS than patients in the corresponding control group.
2. Patients with dementia (aHR = 0.65, 95% CI 0.42–0.98) and those taking doxycycline (aHR = 0.1, 95% CI 0.07–0.15), azithromycin (aHR = 0.07, 95% CI 0.05–0.1), moxifloxacin (aHR = 0.05, 95% CI 0.04–0.08), levofloxacin (aHR = 0.04, 95% CI 0.03–0.06), or ciprofloxacin (aHR = 0.51, 95% CI 0.31–0.83) had a significantly lower risk of CFS than patients in the corresponding control group.

**Table 1:** Characteristics of study participants after propensity score matching

Variable	Potential pathogens				p-value
	No (n = 395,811)		Yes (n = 395,811)		
	n	%	n	%	
Sex					0.1749
Female	231,231	58.42	230,636	58.27	
Male	164,580	41.58	165,175	41.73	
Age, year					< 0.0001
< 40	184,612	46.64	185,276	46.81	
40–65	154,283	38.98	152,370	38.50	
65+	56,916	14.38	58,165	14.70	
Age mean	44.12	16.93	44.18	17.11	0.1710
Comorbidities					
Hypothyroidism	654	0.17	731	0.18	0.0384
Diabetes mellitus	39,642	10.02	39,458	9.97	0.4905
Insomnia	35,966	9.09	36,396	9.20	0.0935
Depression	15,667	3.96	16,355	4.13	0.0001
Anxiety	57,312	14.48	57,795	14.60	0.1236
Dementia	917	0.23	1069	0.27	0.0006
Peptic ulcer	78,871	19.93	79,257	20.02	0.2779
Obesity	2627	0.66	2814	0.71	0.0110
Psoriasis	2224	0.56	2535	0.64	0.0000
Burn	10,722	2.71	11,280	2.85	0.0001
Gout	30,102	7.61	30,276	7.65	0.4613
Dyslipidemia	45,876	11.59	46,019	11.63	0.6158
Inflammatory bowel syndrome	20,993	5.30	21,043	5.32	0.8021
Hepatitis B	9671	2.44	9975	2.52	0.0281
Hepatitis C	2795	0.71	3114	0.79	< 0.0001
Fibromyalgia	78,494	19.83	78,968	19.95	0.1820
Medication					
Doxycycline	2545	0.64	2546	0.64	0.9888
Azithromycin	4807	1.21	4955	1.25	0.1317
Clarithromycin	228	0.06	262	0.07	0.1244
Moxifloxacin	4219	1.07	4379	1.11	0.0827
Levofloxacin	6797	1.72	6999	1.77	0.0827
Ciprofloxacin	344	0.09	364	0.09	0.4521

Chi-square test, t-test

**Table 2:** Cox model with hazard ratios and 95% confidence intervals of Chronic fatigue syndrome associated with pathogens, sex and age

Variable	Chronic fatigue syndrome						
	Event	PY	IR	cHR (95% CI)	p-value	aHR (95% CI)	p-value
Cohort with infection							
No	11,511	3,137,363	3.67	1.00 (reference)	–	1.00 (reference)	–
Yes	21,861	4,047,015	5.40	1.5 (1.46, 1.53)***	0.001	1.5 (1.47, 1.54)***	0.001
Pathogens							
Cohort without infection	11,511	3,137,363	3.67	1.00 (reference)	–	1.00 (reference)	–
Varicella-zoster virus	2382	481,446	4.95	1.36 (1.3, 1.42)***	< 0.001	1.09 (1.04, 1.14)***	< 0.001
Epstein-Barr virus	6	1122	5.35	1.47 (0.66, 3.28)	0.3437	1.71 (0.77, 3.81)	0.1881
* Mycobacterium tuberculosis*	641	111,209	5.76	1.59 (1.47, 1.73)***	< 0.001	1.45 (1.34, 1.57)***	< 0.001
* E. coli*	350	60,035	5.83	1.62 (1.46, 1.8)***	< 0.001	1.17 (1.05, 1.3)**	0.0052
* Candida*	3878	767,616	5.05	1.39 (1.34, 1.44)***	< 0.001	1.43 (1.37, 1.48)***	< 0.001
Enterovirus	29	4396	6.60	1.84 (1.27, 2.64)**	0.0011	1.86 (1.29, 2.67)***	< 0.001
* Salmonella*	137	26,687	5.13	1.42 (1.2, 1.68)***	< 0.001	1.41 (1.19, 1.67)***	< 0.001
* Staphylococcus aureus*	71	11,970	5.93	1.65 (1.3, 2.08)***	< 0.001	1.38 (1.09, 1.74)**	0.0075
* Chlamydia pneumoniae*	3	748	4.01	1.09 (0.35, 3.39)	0.8784	0.93 (0.3, 2.87)	0.8937
Influenza virus	14,219	2,546,888	5.58	1.56 (1.52, 1.6)***	< 0.001	1.67 (1.63, 1.71)***	< 0.001
* Orientia tsutsugamushi*	18	3629	4.96	1.35 (0.85, 2.14)	0.2037	1.38 (0.87, 2.19)	0.1756
* Mycoplasma*	80	21,456	3.73	1.03 (0.82, 1.28)	0.8089	0.93 (0.75, 1.16)	0.5387
Dengue virus	44	9559	4.60	1.32 (0.98, 1.78)	0.0638	1.22 (0.91, 1.64)	0.185
* Borrelia burgdorferi*	3	255	11.77	3.21 (1.04, 9.91)*	0.0425	3.37 (1.09, 10.44)*	0.0355
Sex							
Female	20,794	4,283,422	4.85	1.00 (reference)	–	1.00 (reference)	–
Male	12,578	2,900,956	4.34	0.89 (0.87, 0.91)***	< 0.001	0.91 (0.89, 0.93)***	< 0.001
Age (years)							
< 40	14,080	3,724,544	3.78	1.00 (reference)	–	1.00 (reference)	–
40–64	13,936	2,730,127	5.10	1.35 (1.32, 1.39)***	< 0.001	1.2 (1.17, 1.23)***	< 0.001
65+	5356	729,706	7.34	1.97 (1.91, 2.03)***	< 0.001	1.59 (1.54, 1.65)***	< 0.001

*PY* Person-Year; *IR* Incidence rate, per 1000 persons/years; *HR* Hazard ratio; *CI* confidence interval; *Adjusted HR* adjusted for age, sex, comorbidities and medications in Cox proportional hazards regression

*p < 0.05, **p < 0.01, ***p < 0.001

**Table 3:** Cox model with hazard ratios and 95% confidence intervals of Chronic fatigue syndrome associated with comorbidities

Variable	Chronic fatigue syndrome						
	Event	PY	IR	cHR (95% CI)	p-value	aHR (95% CI)	p-value
Comorbidities							
Hypothyroidism							
No	33,327	7,177,484	4.64	1.00 (reference)	–	1.00 (reference)	–
Yes	45	6894	6.53	1.43 (1.06, 1.91)*	0.0173	1.08 (0.8, 1.44)	0.6134
Diabetes mellitus							
No	29,855	6,687,346	4.46	1.00 (reference)	–	1.00 (reference)	–
Yes	3517	497,032	7.08	1.59 (1.54, 1.65)***	< 0.001	1.05 (1.01, 1.09)*	0.0133
Insomnia							
No	29,282	6,717,076	4.36	1.00 (reference)	–	1.00 (reference)	–
Yes	4090	467,302	8.75	2.01 (1.95, 2.08)***	< 0.001	1.32 (1.27, 1.37)***	< 0.001
Depression							
No	31,603	6,971,899	4.53	1.00 (reference)	–	1.00 (reference)	-
Yes	1769	212,479	8.33	1.83 (1.75, 1.92)***	< 0.001	1.07 (1.01, 1.13)*	0.0126
Anxiety							
No	26,926	6,368,646	4.23	1.00 (reference)	–	1.00 (reference)	–
Yes	6446	815,732	7.90	1.87 (1.81, 1.92)***	< 0.001	1.29 (1.24, 1.33)***	< 0.001
Dementia							
No	33,350	7,180,108	4.64	1.00 (reference)	–	1.00 (reference)	–
Yes	22	4270	5.15	1.2 (0.79, 1.82)	0.3981	0.65 (0.42, 0.98)*	0.0409
Peptic ulcer							
No	25,618	6,086,581	4.21	1.00 (reference)	–	1.00 (reference)	–
Yes	7754	1,097,797	7.06	1.68 (1.63, 1.72)***	< 0.001	1.22 (1.18, 1.25)***	< 0.001
Obesity							
No	33,168	7,149,700	4.64	1.00 (reference)	–	1.00 (reference)	–
Yes	204	34,677	5.88	1.27 (1.1, 1.45)***	< 0.001	1.01 (0.88, 1.16)	0.8833
Psoriasis							
No	33,233	7,153,937	4.65	1.00 (reference)	–	1.00 (reference)	–
Yes	139	30,441	4.57	0.98 (0.83, 1.16)	0.8347	0.85 (0.72, 1)	0.0511
Burn							
No	32,570	7,047,627	4.62	1.00 (reference)	–	1.00 (reference)	–
Yes	802	136,750	5.86	1.27 (1.18, 1.36)***	< 0.001	1.06 (0.99, 1.14)	0.0855
Gout							
No	30,481	6,771,447	4.50	1.00 (reference)	–	1.00 (reference)	–
Yes	2891	412,930	7.00	1.55 (1.49, 1.61)***	< 0.001	1.16 (1.11, 1.21)***	< 0.001
Dyslipidemia							
No	29,181	6,620,858	4.41	1.00 (reference)	–	1.00 (reference)	–
Yes	4191	563,519	7.44	1.7 (1.64, 1.76)***	< 0.001	1.1 (1.06, 1.14)***	< 0.001
Inflammatory bowel syndrome							
No	31,201	6,902,039	4.52	1.00 (reference)	–	1.00 (reference)	–
Yes	2171	282,339	7.69	1.7 (1.63, 1.77)***	< 0.001	1.15 (1.1, 1.21)***	< 0.001
Hepatitis B							
No	32,492	7,052,148	4.61	1.00 (reference)	–	1.00 (reference)	–
Yes	880	132,230	6.66	1.44 (1.34, 1.54)***	< 0.001	1.21 (1.13, 1.3)***	< 0.001
Hepatitis C							
No	32,993	7,148,144	4.62	1.00 (reference)	–	1.00 (reference)	–
Yes	379	36,233	10.46	2.27 (2.06, 2.52)***	< 0.001	1.54 (1.39, 1.71)***	< 0.001
Fibromyalgia							
No	25,843	6,103,927	4.23	1.00 (reference)	–	1.00 (reference)	–
Yes	7529	1,080,451	6.97	1.64 (1.6, 1.69)***	< 0.001	1.29 (1.26, 1.33)***	< 0.001

*PY* Person-Year; *IR* Incidence rate, per 1000 persons/years; *HR* Hazard ratio; *CI* confidence interval; *Adjusted HR* adjusted for age, sex, comorbidities and medications in Cox proportional hazards regression

*p < 0.05, **p < 0.01, ***p < 0.001

**Table 4:** Cox model with hazard ratios and 95% confidence intervals of Chronic fatigue syndrome associated with medications

Variable	Chronic fatigue syndrome						
	Event	PY	IR	cHR (95% CI)	p-value	aHR (95% CI)	p-value
Medication							
Doxycycline							
No	33,349	7,133,590	4.67	1.00 (reference)	–	1.00 (reference)	–
Yes	23	50,788	0.45	0.1 (0.06, 0.15)***	< 0.001	0.1 (0.07, 0.15)***	< 0.001
Azithromycin							
No	33,342	7,087,311	4.70	1.00 (reference)	–	1.00 (reference)	–
Yes	30	97,066	0.31	0.07 (0.05, 0.09)***	< 0.001	0.07 (0.05, 0.1)***	< 0.001
Clarithromycin							
No	33,341	7,178,084	4.64	1.00 (reference)	–	1.00 (reference)	–
Yes	31	6294	4.93	1.05 (0.74, 1.49)	0.7818	1 (0.7, 1.42)	0.9838
Moxifloxacin							
No	33,350	7,099,137	4.70	1.00 (reference)	–	1.00 (reference)	–
Yes	22	85,241	0.26	0.06 (0.04, 0.08)***	< 0.001	0.05 (0.04, 0.08)***	< 0.001
Levofloxacin							
No	33,346	7,048,173	4.73	1.00 (reference)	–	1.00 (reference)	–
Yes	26	136,205	0.19	0.04 (0.03, 0.06)***	< 0.001	0.04 (0.03, 0.06)***	< 0.001
Ciprofloxacin							
No	33,356	7,176,747	4.65	1.00 (reference)	–	1.00 (reference)	–
Yes	16	7630	2.10	0.45 (0.28, 0.74)**	0.0015	0.51 (0.31, 0.83)**	0.0071

*PY* Person-Year; *IR* Incidence rate, per 1000 persons/years; *HR* Hazard ratio; *CI* confidence interval; *Adjusted HR* adjusted for age, sex, comorbidities and medications in Cox proportional hazards regression

*p < 0.05, **p < 0.01, ***p < 0.001

Table 5 reveals that irrespective of sex, age, comorbidities, and medications, patients with a potential pathogen had a higher risk of CFS than those without the potential pathogens.

**Table 5:** Incidence rates, hazard ratios and confidence intervals of patients with infection associated with risk factors

Variable	Potential pathogens						cHR (95% CI)	p-value	aHR (95% CI)	p-value
	No			Yes						
	Event	PY	IR	Event	PY	IR				
Sex										
Female	7317	1,866,947	3.92	13,477	2,416,475	5.58	1.45 (1.4, 1.49)***	< 0.001	1.45 (1.41, 1.5)***	< 0.001
Male	4194	1,270,416	3.30	8384	1,630,540	5.14	1.59 (1.53, 1.65)***	< 0.001	1.59 (1.54, 1.65)***	< 0.001
Age (years)										
< 40	4855	1,615,644	3.00	9225	2,108,900	4.37	1.46 (1.41, 1.51)***	< 0.001	1.46 (1.41, 1.51)***	< 0.001
40–64	4609	1,187,150	3.88	9327	1,542,978	6.04	1.59 (1.53, 1.64)***	< 0.001	1.59 (1.53, 1.65)***	< 0.001
65+	2047	334,569	6.12	3309	395,137	8.37	1.39 (1.32, 1.47)***	< 0.001	1.41 (1.34, 1.49)***	< 0.001
Comorbidities										
Hypothyroidism										
No	11,492	3,134,447	3.67	21,835	4,043,037	5.40	1.5 (1.47, 1.53)***	< 0.001	1.51 (1.47, 1.54)***	< 0.001
Yes	19	2916	6.52	26	3978	6.54	1.01 (0.56, 1.84)	0.963	0.92 (0.5, 1.69)	0.7866
Diabetes mellitus										
No	10,126	2,900,216	3.49	19,729	3,787,130	5.21	1.51 (1.48, 1.55)***	< 0.001	1.51 (1.47, 1.55)***	< 0.001
Yes	1385	237,147	5.84	2132	259,885	8.20	1.44 (1.35, 1.54)***	< 0.001	1.45 (1.36, 1.55)***	< 0.001
Insomnia										
No	9926	2,920,880	3.40	19,356	3,796,197	5.10	1.52 (1.48, 1.55)***	< 0.001	1.52 (1.48, 1.55)***	< 0.001
Yes	1585	216,483	7.32	2505	250,818	9.99	1.41 (1.32, 1.5)***	< 0.001	1.41 (1.32, 1.5)***	< 0.001
Depression										
No	10,852	3,041,160	3.57	20,751	3,930,739	5.28	1.5 (1.47, 1.54)***	< 0.001	1.51 (1.47, 1.54)***	< 0.001
Yes	659	96,203	6.85	1110	116,276	9.55	1.43 (1.3, 1.58)***	< 0.001	1.43 (1.3, 1.58)***	< 0.001
Anxiety										
No	9055	2,764,969	3.27	17,871	3,603,677	4.96	1.53 (1.49, 1.57)***	< 0.001	1.53 (1.49, 1.57)***	< 0.001
Yes	2456	372,394	6.60	3990	443,338	9.00	1.41 (1.34, 1.48)***	< 0.001	1.41 (1.34, 1.48)***	< 0.001
Dementia										
No	11,499	3,134,988	3.67	21,851	4,045,120	5.40	1.5 (1.47, 1.53)***	< 0.001	1.5 (1.47, 1.54)***	< 0.001
Yes	12	2375	5.05	10	1895	5.28	1.06 (0.46, 2.47)	0.8917	1.14 (0.48, 2.72)	0.7648
Peptic ulcer										
No	8594	2,632,611	3.26	17,024	3,453,971	4.93	1.52 (1.48, 1.56)***	< 0.001	1.52 (1.48, 1.56)***	< 0.001
Yes	2917	504,752	5.78	4837	593,044	8.16	1.45 (1.38, 1.52)***	< 0.001	1.46 (1.39, 1.53)***	< 0.001
Obesity										
No	11,422	3,121,792	3.66	21,746	4,027,908	5.40	1.5 (1.47, 1.54)***	< 0.001	1.51 (1.47, 1.54)***	< 0.001
Yes	89	15,571	5.72	115	19,107	6.02	1.07 (0.81, 1.42)	0.6106	0.99 (0.75, 1.31)	0.9581
Psoriasis										
No	11,470	3,123,972	3.67	21,763	4,029,965	5.40	1.5 (1.46, 1.53)***	< 0.001	1.5 (1.47, 1.54)***	< 0.001
Yes	41	13,391	3.06	98	17,050	5.75	1.96 (1.36, 2.83)***	< 0.001	1.9 (1.32, 2.75)***	< 0.001
Burn										
No	11,249	3,075,224	3.66	21,321	3,972,404	5.37	1.49 (1.46, 1.53)***	< 0.001	1.5 (1.47, 1.53)***	< 0.001
Yes	262	62,139	4.22	540	74,611	7.24	1.77 (1.53, 2.05)***	< 0.001	1.71 (1.48, 1.99)***	< 0.001
Gout										
No	10,394	2,943,302	3.53	20,087	3,828,145	5.25	1.51 (1.47, 1.54)***	< 0.001	1.51 (1.47, 1.54)***	< 0.001
Yes	1117	194,061	5.76	1774	218,869	8.11	1.45 (1.35, 1.57)***	< 0.001	1.45 (1.35, 1.57)***	< 0.001
Dyslipidemia										
No	9927	2,872,979	3.46	19,254	3,747,879	5.14	1.5 (1.47, 1.54)***	< 0.001	1.51 (1.47, 1.54)***	< 0.001
Yes	1584	264,384	5.99	2607	299,136	8.72	1.49 (1.4, 1.59)***	< 0.001	1.49 (1.4, 1.58)***	< 0.001
Inflammatory bowel disease										
No	10,688	3,008,426	3.55	20,513	3,893,613	5.27	1.51 (1.47, 1.54)***	< 0.001	1.51 (1.48, 1.55)***	< 0.001
Yes	823	128,937	6.38	1348	153,402	8.79	1.41 (1.3, 1.54)***	< 0.001	1.41 (1.3, 1.54)***	< 0.001
Hepatitis B										
No	11,158	3,076,681	3.63	21,334	3,975,467	5.37	1.5 (1.47, 1.54)***	< 0.001	1.51 (1.48, 1.55)***	< 0.001
Yes	353	60,682	5.82	527	71,548	7.37	1.3 (1.13, 1.49)***	< 0.001	1.27 (1.11, 1.46)***	< 0.001
Hepatitis C										
No	11,361	3,120,762	3.64	21,632	4,027,383	5.37	1.5 (1.47, 1.54)***	< 0.001	1.51 (1.47, 1.54)***	< 0.001
Yes	150	16,601	9.04	229	19,632	11.66	1.33 (1.08, 1.63)**	0.0074	1.32 (1.07, 1.62)**	0.0088
Fibromyalgia										
No	8622	2,640,127	3.27	17,221	3,463,800	4.97	1.53 (1.49, 1.57)***	< 0.001	1.53 (1.49, 1.57)***	< 0.001
Yes	2889	497,236	5.81	4640	583,214	7.96	1.41 (1.34, 1.47)***	< 0.001	1.41 (1.35, 1.48)***	< 0.001

*PY* Person-Year; *IR* Incidence rate, per 1000 persons/years; *HR* Hazard ratio; *CI* confidence interval; *Adjusted HR* adjusted for age, sex, comorbidities and medications in Cox proportional hazards regression

*p < 0.05, **p < 0.01, ***p < 0.001

## Discussion

Our findings indicate that infections with various pathogens—bacteria, viruses, and fungi—were associated with an increased incidence of CFS (Table 2, Fig. 2). Only *ICD* codes for infections were included in our data; codes of colonization were excluded from our analysis. The hazard ratios of CFS were positively correlated with age; older adults were more likely to develop CFS after an episode of infection (Table 2). Moreover, infection increased the incidence of CFS in most of the comorbidity subgroups, but not in all of them (Table 5). However, the mechanisms behind the variation in incidence among different age groups and comorbidity subgroups were warranted for further investigations.

The study found that typical bacteria, including those that are intracellular (i.e., *Salmonella*), extracellular (i.e., *E. coli*) and with both properties (i.e., *Staphylococcus aureus*) were associated with an increased incidence of CFS. Only a few studies have addressed this topic. In 2007, Maes et al. found that serum immunoglobulin A and M against lipopolysaccharides of enterobacteria—such as *Pseudomonas aeruginosa*, *Proteus mirabilis* and *Klebsiella pneumonia—*were elevated in patients with CFS [38]. Typical bacteria have been indicated to influence the human immune system. For example, *Staphylococcus* is known for its immunomodulation ability, which is achieved by affecting T cells [39]. However, there was no other direct evidence studying the association of staphylococcal infection and CFS.

The study found that influenza with H1N1 influenza virus was associated with an increased incidence of CFS [40]. In our study, we also observed an association between influenza and CFS, but it was not limited to H1N1. Several studies have verified, mostly through serological approaches, that EBV infection increases the risk of developing CFS [41, 42]. However, in the present study, the associations of EBV and CMV with CFS were considered nonsignificant due to the limited number of identified cases. Similarly, significance could not be established for several other pathogens with limited cases, including *Chlamydia pneumoniae*, *Mycoplasma*, dengue virus, *Orientia tsutsugamushi*, and *Borrelia burgdorferi*.

Studies involving multiple cohorts and cross-sectional studies have reported similarities between the symptoms of long COVID and CFS, such as cognitive impairment and fatigue over a follow-up duration ranging from 12 weeks to 6 months [43, 44]. Similar to CFS, long COVID presents with IL-6 dysregulation and disrupted T cell responses [45]. Moreover, elevated CD8+ T cells and increased type 1 cytokines were linked to abnormal chest X-ray findings in patients who had had COVID-19 six months after their discharge from the hospital [46]. Although the exact mechanisms of long COVID and CFS are not fully understood, these overlapping features warrant future research exploring both CFS and long COVID.

Candida albicans in fecal microflora was previously observed in patients with CFS [47]. Chronic Intestinal Candidiasis was indicated to be one of the possible factors of CFS and nutritional therapy for candidiasis, like an anti-candida diet and natural antifungals (i.e., caprylic acid), has shown to reduce symptoms of CFS [48, 49]. Scientists are increasingly recognizing the role of the gut–brain axis in patients with CFS. Multiple pathways have been proposed to explain gut–brain communication, such as the immune system (i.e., cytokines), hormones (i.e., gamma-aminobutyric acid), the neuron system (i.e., Vagus nerve), and metabolites (i.e., short-chain fatty acids) [50]. An altered gut microbiome composition is associated with not only CFS but a variety of diseases, including inflammatory bowel disease, multiple sclerosis, and systemic lupus erythematosus [51–53]. With regard to other fungi, mycotoxins detected in the urine of patients with CFS raised concern over the role of mycotoxin-producing mold, such as *Aspergillus*, in CFS [54]. Since research focusing on the association of CFS and fungi other than Candida is scarce, this topic deserves more attention.

Our finding indicated that the use of doxycycline, azithromycin, moxifloxacin, ciprofloxacin, or levofloxacin was significantly associated with decreased incidence of CFS (Tables 2 and 4). The exact mechanism underlying how these antibiotics prevent CFS is unknown, but they influence the immune system in different ways. Azithromycin inhibits transcription factors and their downstream inflammatory cytokines, such as the PI3K/AKT/NF-ҡB, ERK1/2/NF-ҡB, and AP-1 pathways [55, 56]. One study revealed that fluoroquinolones increased production of anti-inflammatory cytokines such as TGF-β and IL-10 and decreased that of proinflammatory cytokines such as IL-1β, IL-6, and TNF-α [31]. In addition to inhibiting TNF-α, doxycycline exerts immunomodulatory effects by downregulating proinflammatory enzymes, such as nitric oxide synthetase and matrix metalloproteinases [32, 33, 57]. A randomized controlled trial revealed that long-term doxycycline treatment offered no benefit in reducing fatigue in patients with Q Fever fatigue syndrome [58].

Our study, despite its limitations, provides significant insights into the association between CFS and infections. One limitation was the potential impact of rare pathogens on CFS due to the limited numbers of cases. However, our primary objective was to investigate the association between CFS and most seen pathogens, which we successfully demonstrated. Another limitation was our inability to conduct real-time evaluations of each infectious disease’s severity due to the unavailability of patients’ vital signs and laboratory data in the NHIRD. However, we accounted for comorbidities and adjusted for confounding factors to measure the risk of CFS-related factors.

Lastly, due to data anonymity, we couldn’t access information on renal or hepatic dose adjustments of antibiotics for each patient. Nevertheless, we presented most relevant pathogens that might cause CFS and considered comorbidities, which allowed us to adjust the hazard ratio accordingly.

Despite these limitations, our study has notable strengths. It includes a large number of cases and controls, making it the first one to demonstrate the association between CFS and most commonly seen pathogens using a big database. Our findings challenge previous beliefs that only atypical bacteria and viruses are associated with CFS by revealing that typical bacteria can also be linked to CFS. This supports the theory that pathogens play a “triggering” role in CFS.

In conclusion, our study indicates a higher risk of CFS following common infections, underscoring the triggering role of infection in CFS. Interestingly, we found that the incidence of CFS was lower when patients took antibiotics with immunomodulatory properties. This finding could shed light on potential treatment strategies for CFS.

## Data Availability

The data that support the findings of this study are available from the NHIRD. Restrictions apply to the availability of these data, which were used under license for this study. Data are available with the permission directly from the NHIRD.
